# Medicinal Plants for Skin Disorders: Phytochemistry and Pharmacological Insights

**DOI:** 10.3390/molecules30153281

**Published:** 2025-08-06

**Authors:** Nazerke Bolatkyzy, Daniil Shepilov, Rakhymzhan Turmanov, Dmitriy Berillo, Tursunay Vassilina, Nailya Ibragimova, Gulzat Berganayeva, Moldyr Dyusebaeva

**Affiliations:** 1 Faculty of Chemistry and Chemical Technology, Al-Farabi Kazakh National University, 71 Al-Farabi Avenue, Almaty 050042, Kazakhstan; nbolatkyzy98@gmail.com (N.B.);; 2 Department of Chemistry, Abai Kazakh National Pedagogical University, Dostyk Avenue 13, Almaty 050010, Kazakhstan; r.turmanov@abaiuniversity.edu.kz; 3 Center of Agro Competence, M. Kozybayev North-Kazakhstan University, Petropavlovsk 150000, Kazakhstan; daberillo@ku.edu.kz; 4 Kazakh National Agrarian Research University, St. Named After Valikhanov 137, Almaty 050000, Kazakhstan; v_tursunai@mail.ru; 5 Faculty of Engineering and Information Technologies, Kazakh-German University (DKU), Almaty 050010/A26C7F8, Kazakhstan; nailya.73@mail.ru

**Keywords:** medicinal plants, phytochemistry, skin diseases, flavonoids, anthocyanins, sesquiterpenes, antioxidant activity, anti-inflammatory effect, traditional medicine, bioactive compounds

## Abstract

Skin disorders are common and often chronic conditions with significant therapeutic challenges. Limitations of conventional treatments, such as adverse effects and antimicrobial resistance, have increased interest in plant-based alternatives. This article presents the phytochemical composition and pharmacological potential of several medicinal plants traditionally used in the treatment of skin diseases, including *Rubus vulgaris*, *Plantago major*, *Artemisia terrae-albae*, and *Eryngium planum*. Based on an analysis of scientific literature, the presence of bioactive compounds—including flavonoids, anthocyanins, phenolic acids, tannins, and sesquiterpenes—is summarized, along with their antioxidant, anti-inflammatory, and antimicrobial effects. Emphasis is placed on the correlation between traditional ethnomedicinal applications and pharmacological mechanisms. The findings support the potential of these species as sources for dermatological phytotherapeutics. Further research is needed to standardize active constituents, assess safety, and conduct clinical validation.

## 1. Introduction

Skin disorders are among the most prevalent health conditions worldwide and significantly affect patients’ quality of life. According to the European Academy of Dermatology and Venereology, up to 43% of the adult population in Europe suffers from at least one dermatological condition, including eczema, acne, psoriasis, dermatitis, and fungal skin infections [1]. These conditions are often accompanied by inflammation, itching, and impaired skin barrier function, and frequently manifest as chronic diseases. Moreover, they may lead to considerable psychological and social discomfort, particularly in cases of prolonged progression and visible cosmetic defects [2].

Modern dermatology offers a wide range of synthetic treatments, from topical corticosteroids to systemic immunomodulators. However, long-term use of these agents is frequently associated with adverse effects such as skin atrophy, hypopigmentation, resistance development, and microbiome disruption [3]. As a result, there is growing interest in the use of herbal medicines—plant-based therapeutics known for their mild action, favorable tolerability, and broad pharmacological effects [4].

Medicinal plants have been used for centuries in traditional healing systems such as Ayurveda, Traditional Chinese Medicine, European phytotherapy, and East Slavic folk medicine for managing various skin conditions, including ulcers, burns, eczema, and inflammatory dermatoses [5,6]. With the accumulation of scientific evidence on the chemical composition and mechanisms of action of plant extracts, their use has been increasingly integrated into evidence-based medicine. Many plants that were once used empirically are now included in officially registered dermatological and cosmeceutical formulations listed in international pharmacopoeias [7].

Plants possess a unique biochemical potential, including flavonoids, terpenoids, polysaccharides, phenolic acids, tannins, and essential oils, which exhibit a broad spectrum of biological activities relevant to skin health [8,9]. These phytocompounds have demonstrated the ability to modulate pro-inflammatory cytokine expression, inhibit key inflammatory enzymes such as cyclooxygenase-2 (COX-2), promote epidermal regeneration, and restore skin barrier integrity [10,11]. Such mechanisms are particularly relevant in the context of multifactorial dermatological conditions like acne vulgaris and psoriasis. The pathogenesis of acne involves hyperseborrhea, follicular hyperkeratinization, *Cutibacterium acnes* colonization, and cytokine-mediated inflammation (e.g., IL-1β, TNF-α), while psoriasis is characterized by Th1/Th17-mediated immune dysregulation, overexpression of IL-17, IL-23, and interferon-γ and keratinocyte hyperproliferation. These pathological mechanisms represent suitable therapeutic targets for phytochemicals exhibiting antioxidant, anti-inflammatory, antimicrobial, and immunomodulatory effects.

This review article presents a comprehensive analysis of medicinal plants used in the treatment of dermatological conditions, with a focus on their phytochemical composition, mechanisms of action, and recent pharmacological evidence. More than 30 plant species traditionally used in both folk and official medicine for managing eczema, dermatitis, psoriasis, burns, acne, trophic ulcers, and other skin disorders are discussed. This article also highlights the potential of integrating herbal remedies into dermatological and cosmeceutical formulations. Special attention is given to species used in Central Asian traditional medicine, selected based on historical usage combined with modern experimental validation of biological activities. The objective of this review is to synthesize existing knowledge on these species in the context of current pharmacological research and identify their translational potential for evidence-based dermatology.

## 2. Methodology of Literature Selection

The selection of plant species for this review was based on a combined ethnobotanical and pharmacological approach, with a particular focus on the traditional medical systems of Central Asia. Initial inclusion criteria prioritized plants historically used in regional ethnomedicine for the treatment of skin disorders such as wounds, burns, ulcers, dermatitis, eczema, and acne. Ethnopharmacological relevance was established through analysis of classical sources and field ethnobotanical surveys, with attention given to species showing consistent topical application across cultures and time periods.

Studies were included if they demonstrated skin-related activity in vitro or in vivo, including anti-inflammatory, antimicrobial, antioxidant, or regenerative effects. Older sources were used for historical or phytochemical background, while recent literature supported mechanistic and pharmacological analysis.

To maintain clarity and avoid redundancy, only one representative compound was selected for each plant species. These compounds were chosen based on either their quantitative predominance in the plant material or the frequency of their pharmacological assessment in the context of skin-related bioactivity. While each species may contain multiple active constituents, including structurally related compounds, this review focuses on those with the most evidence-based dermatological relevance.

The final list comprised twenty species selected for their documented ethnomedical use, presence of bioactive compounds relevant to skin health, and availability of modern experimental evidence. This methodology ensured that both traditional knowledge and contemporary scientific data were integrated to assess the dermatological potential of the selected medicinal plants.

## 3. Medicinal Plants

### 3.1. Bergenia crassifolia

*Bergenia crassifolia*, a perennial herbaceous plant belonging to the family *Saxifragaceae*, is native to Siberia, the Altai Mountains, Mongolia, China, and Korea. It typically grows on rocky slopes, screes, and within coniferous and deciduous forests, preferring shaded and moist habitats [12].

**Figure 1:**
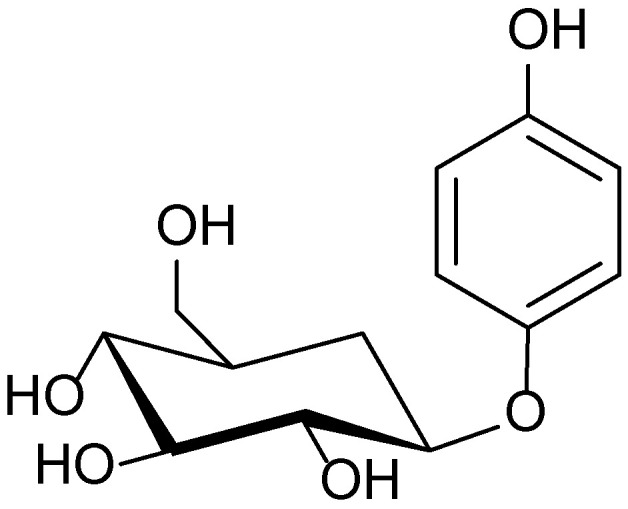
Structural formula of arbutin—the main bioactive phenolic glycoside of *Bergenia crassifolia*.

Phytochemical investigations of species within the *Bergenia* genus, including *B. crassifolia* and *B. cordifolia*, have revealed a high content of arbutin, flavonoids, hydroxycinnamic acids, polyphenols, and tannins—compounds known for their dermatological activity. Arbutin (Figure 1), the principal bioactive constituent of these plants, exerts a pronounced depigmenting effect by inhibiting tyrosinase, a key enzyme involved in melanin biosynthesis. High-performance liquid chromatography with ultraviolet detection (HPLC-UV) analysis showed that the arbutin content in the leaves of various *Bergenia* specimens ranged from 4.8 to 9.8 g/100 g, with the highest levels reported in samples collected from Finland.

**Figure 2:**
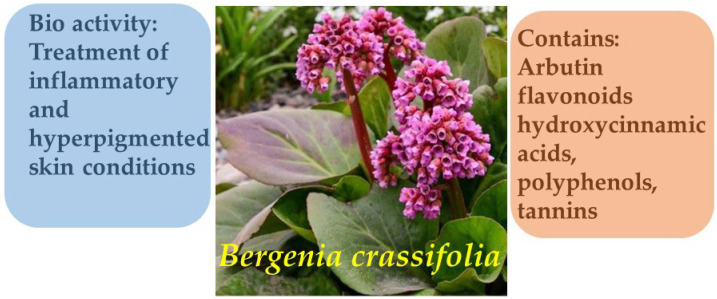
Phytotherapeutic application and chemical composition of *Bergenia crassifolia*.

In addition, substantial concentrations of polyphenolic compounds (up to 9.27 g/100 g), tannins (up to 6.7 g/100 g), and hydroxycinnamic acids (up to 2.42 g/100 g) underscore the potential therapeutic relevance of *Bergenia* species in the treatment of inflammatory and hyperpigmented skin conditions. The co-presence of arbutin and other phenolic constituents suggests a possible synergistic interaction, potentially enhancing the overall dermatological efficacy of *Bergenia*-based formulations (Figure 2) [13,14,15].

A study published in the *Mongolian Journal of Chemistry* investigated the wound-healing activity of a composition based on a hydrogel of poly(hexamethylene guanidine) hydrochloride combined with an extract of *Bergenia crassifolia* in a thermal burn model on laboratory animals. The results demonstrated that this formulation contributes to the normalization of antioxidant activity and leukocyte levels. Morphological analysis revealed that wound healing was significantly accelerated in the treatment group compared to the control, as evidenced by a reduction in the thickness of the leukocyte-necrotic eschar, enhanced epithelialization, and complete closure of the skin defect [16].

*Bergenia crassifolia* is also utilized in cosmetic products aimed at pore tightening, reducing oily skin shine, and overall improvement of skin condition. Decoctions and infusions prepared from its leaves and rhizomes are commonly applied in the form of lotions, facial rinses, and compresses. In clinical settings, *Bergenia*-based formulations are used in the treatment of burns and trophic ulcers as part of combination therapy, promoting epithelial regeneration and protecting damaged tissue from microbial infection [17,18,19].

### 3.2. Black Elderberry (Sambucus nigra)

*Sambucus nigra* is a perennial shrub belonging to the family *Adoxaceae*, widely distributed in the temperate regions of Europe, North Africa, and Western Asia. Its flowers and fruits have been traditionally and officially employed in medicine, including in dermatology and cosmetology applications [20,21].

**Figure 3:**
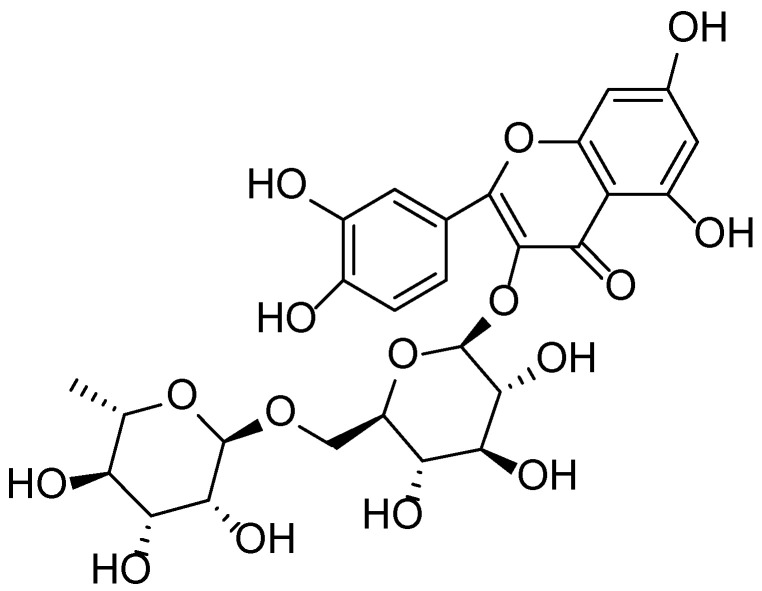
Structural formula of rutin—a key flavonoid component found in *Sambucus nigra* flowers and *Solidago canadensis*.

The flowers of *S. nigra* contain flavonoids (notably rutin (Figure 3) and quercetin), essential oils, organic acids (such as malic, acetic, and valeric acids), tannins, and mucilaginous compounds. The fruits are rich in anthocyanins, vitamin C, B-complex vitamins, sugars, pectins, and carotenoids. Due to this diverse phytochemical profile, *Sambucus*-based preparations exhibit antioxidant, anti-inflammatory, bactericidal, and anti-edematous properties [22,23,24].

In a study by [25], the anti-inflammatory properties of *Sambucus nigra* leaf extracts were investigated. The results demonstrated that the extracts suppressed the secretion of tumor necrosis factor-alpha (TNF-α) and reduced the production of reactive oxygen species (ROS) in lipopolysaccharide (LPS)-stimulated neutrophils. These findings highlight the anti-inflammatory potential of *S. nigra* leaves, indicating their relevance as a botanical source for treating inflammatory skin conditions.

Berry extracts of *Sambucus nigra* obtained by supercritical fluid extraction exhibited promising potential for the prevention and treatment of dermatological disorders, primarily through the inhibition of collagenase and elastase—key enzymes involved in the degradation of the extracellular skin matrix. The most potent inhibitory activity was observed in the extract derived from dried berries using absolute ethanol, with collagenase inhibition reaching 84.7%, comparable to the positive control (white tea extract). Additionally, the same extract demonstrated high elastase inhibitory activity (77.3%), exceeding that of the reference compound ursolic acid. As these enzymes are critically involved in skin aging, inflammation, and loss of elasticity, the findings underscore the dermatological significance of *Sambucus nigra* extracts.

Moreover, these extracts exhibited antioxidant properties and were successfully encapsulated in PLGA-based polymeric nanoparticles, which improved their stability and potential skin permeability. The resulting nanostructures maintained their bioactivity and exhibited a skin-compatible pH profile, supporting their potential application in dermatological and cosmetic formulations [26].

Fruit extracts of *Sambucus nigra* also demonstrated strong anti-photoaging and anti-inflammatory effects in a UVB-induced human keratinocyte (HaCaT) damage model. Treatment with the extract significantly reduced ROS production, and inhibited the expression of matrix metalloproteinase-1 (MMP-1), interleukin-6 (IL-6), and vascular endothelial growth factor (VEGF), suggesting a protective effect against collagen degradation and inflammation. The underlying mechanisms included inhibition of the MAPK/AP-1 and NF-κB pathways, as well as activation of the Nrf2/HO-1 antioxidant system and the TGF-β/Smad signaling cascade responsible for type I procollagen synthesis. These results suggest that *Sambucus nigra* is a promising botanical agent for the prevention and treatment of photoaging and UV-induced inflammatory skin conditions [27].

### 3.3. Broadleaf Plantain (Plantago major)

*Plantago major* L., a member of the Plantaginaceae family, is a perennial herbaceous plant widely distributed across temperate and subtropical regions of Europe, Asia, and the Americas. It commonly inhabits anthropogenic environments such as roadsides, wastelands, pastures, and disturbed landscapes, reflecting its high adaptability to adverse ecological conditions [28,29,30].

**Figure 4:**
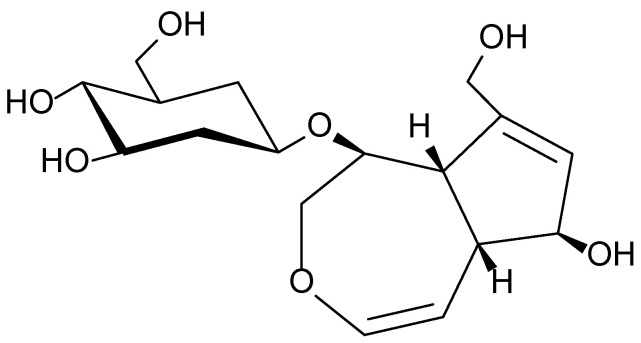
Structural formula of aucubin, the main bioactive iridoid glycoside found in *Plantago major*.

The phytochemical composition of *Plantago major* is characterized by a wide range of biologically active compounds, including phenolic acids (notably chlorogenic and ferulic acids), iridoid glycosides (aucubin and catalpol), flavonoids (apigenin and luteolin), polysaccharides, tannins, and saponins. Recent studies have confirmed the plant’s antioxidant, anti-inflammatory, antibacterial, and wound-healing activities, which are attributed to these constituents. Of particular interest is aucubin (Figure 4), a compound with pronounced biological activity that inhibits pro-inflammatory cytokines and promotes tissue regeneration [31,32,33].

In traditional medicine across Europe and Central Asia, the leaves of *Plantago major* are used to treat wounds, burns, ulcers, skin irritations, as well as respiratory and digestive disorders. In Traditional Chinese Medicine (TCM), the plant is recognized for its ability to clear internal heat, promote wound healing, and reduce inflammation. Ethnobotanical studies conducted in Russia and Kazakhstan have documented the widespread use of *Plantago major* in the form of decoctions, infusions, and topical ointments for the treatment of various skin conditions and wounds, supporting its potential as a promising ingredient in dermatological preparations [34,35].

### 3.4. Canadian Goldenrod (Solidago canadensis)

*Solidago canadensis* is a perennial herbaceous plant belonging to the Asteraceae family, widely distributed across North America and introduced to Europe and Asia. In both traditional and official medicine, the aerial parts of the plant are used, being rich in biologically active constituents.

**Figure 5:**
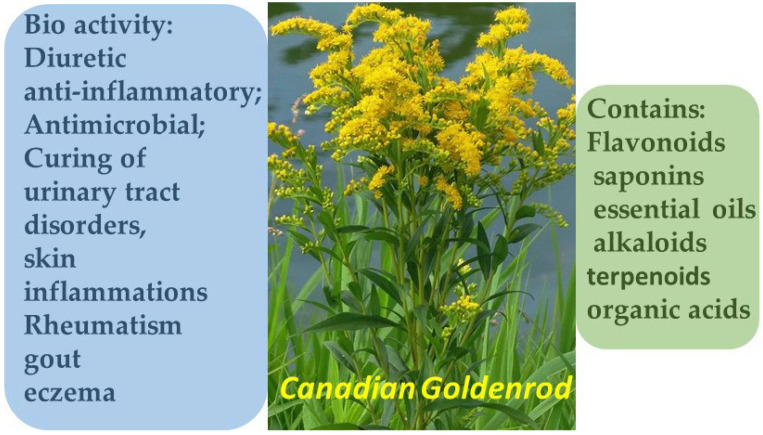
Phytotherapeutic application and chemical composition of *Solidago canadensis*.

Solidago canadensis is characterized by a diverse phytochemical composition, including flavonoids, saponins, terpenoids, phenolic acids, essential oils, and alkaloids. The presence of these constituents underlies its investigated pharmacological activities—anti-inflammatory, antimicrobial, and diuretic effects [36,37]. In European traditional medicine, it has been used for over 700 years, particularly in the treatment of urinary tract disorders, skin inflammations, rheumatism, gout, and eczema. Contemporary studies confirm that *S. canadensis* extracts exhibit antibacterial, antiseptic, analgesic, diuretic, anti-inflammatory, and antioxidant effects (Figure 5). In particular, the plant’s essential oils have demonstrated activity against pathogenic fungi (*Botrytis cinerea*), Gram-positive bacteria, and *Salmonella*, as well as potent insect-repellent properties.

Flavonoids and phenolic compounds isolated from the plant show strong free radical scavenging capacity, positioning *Solidago canadensis* as a promising candidate for the development of phytotherapeutic and cosmetic products aimed at skin restoration, inflammation control, and prevention of age-related skin changes [38].

The leaves of *Solidago canadensis* are rich in a complex array of phenolic compounds, among which rutin (quercetin-3-*O*-rutinoside) (Figure 3) is the predominant flavonoid, known for its potent antioxidant and anti-inflammatory properties. Studies have shown that rutin and other flavonoid glycosides are capable of forming complexes with ammonium ions, resulting in novel polar compounds with notable biological activity.

These complexes exhibit stimulatory effects on root growth in soybean and chrysanthemum seedlings, particularly promoting lateral root formation, suggesting potential applications in phytotherapy and cosmetology for enhancing skin regeneration. Additionally, these compounds have been shown to induce positive chemotaxis in symbiotic bacteria such as *Pseudomonas putida*, indicating a microbiome-modulating effect that may enhance the skin’s defense mechanisms. At low concentrations (up to 20 µg/mL), these complexes display biostimulant activity; however, when the concentration exceeds 100 µg/mL, they begin to inhibit growth, likely due to allelopathic effects [39].

### 3.5. Coltsfoot (Tussilago farfara)

*Tussilago farfara* L., a member of the Asteraceae family, is a widely distributed perennial plant commonly found in temperate zones of Eurasia, including Europe, the Caucasus, Siberia, and the Russian Far East. According to research, in the southern regions of Primorsky Krai it predominantly grows along roadsides, in vacant lots, abandoned orchards, and other anthropogenically disturbed habitats, indicating a tendency for naturalization primarily in artificially transformed ecosystems. At the same time, the plant is actively cultivated and harvested in Bulgaria, where its raw material is used for the analysis of biologically active compounds, highlighting the persistent presence of *T. farfara* in both pharmaceutical practices and the regional flora [40,41].

**Figure 6:**
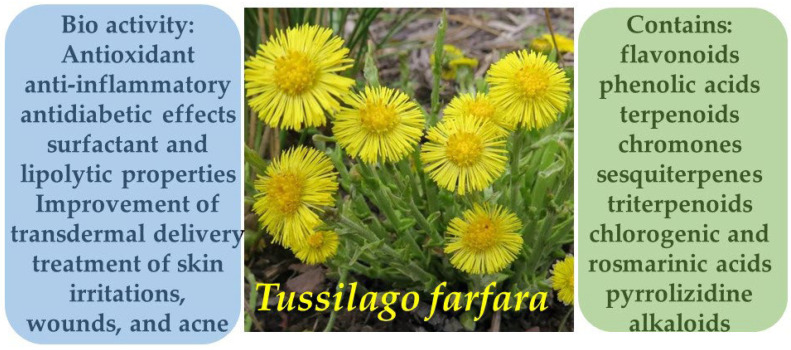
Phytotherapeutic application and chemical composition of *Tussilago farfara*.

**Figure 7:**
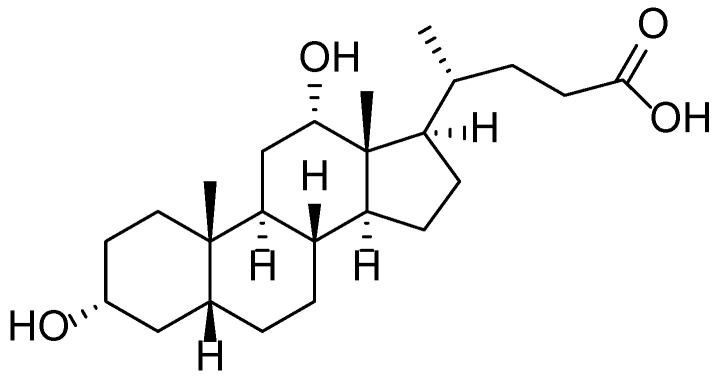
Structural formula of deoxycholic acid—a secondary bile acid with surfactant and lipolytic properties used in pharmaceutical and cosmetic formulations.

The chemical composition of *Tussilago farfara* L. is notably complex and diverse, comprising flavonoids, phenolic acids, terpenoids, alkaloids, and chromones. Contemporary studies have identified over 150 compounds in the plant’s leaves, with particularly active constituents including derivatives of dicaffeoylquinic acid (e.g., compounds 7-12), which exhibit significant inhibitory activity against aldose reductase—an enzyme associated with diabetic complications [42]. Moreover, novel flavonoid glycosides have been isolated, such as kaempferol 3-*O*-[3,4-*O*-(isopropylidene)-α-l-arabinopyranoside], reported for the first time in this species. Additional constituents identified in the leaves and floral buds include sesquiterpenes, triterpenoids, chromones, chlorogenic and rosmarinic acids, as well as pyrrolizidine alkaloids, some of which are known to possess toxic properties. This extensive array of secondary metabolites underpins the pharmacological activity of the plant, including its antioxidant, anti-inflammatory, and antidiabetic effects (Figure 6) [43]. In modern formulations, these compounds may be combined with auxiliary agents such as deoxycholic acid—a secondary bile acid with surfactant and lipolytic properties—to improve transdermal delivery and efficacy in pharmaceutical and cosmetic products (Figure 7).

*Tussilago farfara* L. has long been utilized in both European and Asian traditional medicine for the treatment of dermatological and respiratory disorders. In Traditional Chinese Medicine, the floral buds (*Farfarae flos*) are primarily used due to their expectorant, anti-inflammatory, and soothing properties, which also extend to the treatment of skin irritations, wounds, and acne. Phytochemical studies have confirmed that the major bioactive compounds in the plant include flavonoids, phenolic acids, and sesquiterpenes—particularly tussilagone—which exhibit antioxidant, antimicrobial, and wound-healing activities. In Ayurvedic practice, the plant is also used topically for inflammatory skin conditions and to promote tissue regeneration. Recent studies suggest that *T. farfara* demonstrates significant anti-inflammatory effects by suppressing the expression of pro-inflammatory cytokines and inhibiting key enzymes involved in skin inflammation [44,45].

In Belarusian folk medicine, *Tussilago farfara* L. has been widely employed topically for skin inflammations, headaches, and wound treatment. Ethnobotanical fieldwork conducted in the Lyuban district documented the use of leaves and flowers in the form of infusions, decoctions, and fresh applications for treating wounds and inflamed skin. Local residents applied compresses of fresh leaves directly to affected areas and also used infusions both internally and externally to accelerate healing and relieve pain. These findings highlight the enduring use of *T. farfara* in Eastern European ethnomedicine as an accessible and effective remedy for inflammatory skin conditions, supporting its potential inclusion in dermatological phytopharmaceuticals [46].

### 3.6. Common Blackberry (Rubus vulgaris)

*Rubus vulgaris* Weihe & Nees, a member of the Rosaceae family, is a perennial shrub widely distributed across temperate regions of Europe, Asia, and North America. The plant typically grows along forest edges, in shrublands, along roadsides, and in disturbed habitats, demonstrating a high degree of adaptability to diverse ecological conditions. In Kazakhstan, *Rubus vulgaris* is commonly found in foothill and forest-steppe zones [47,48,49,50].

**Figure 8:**
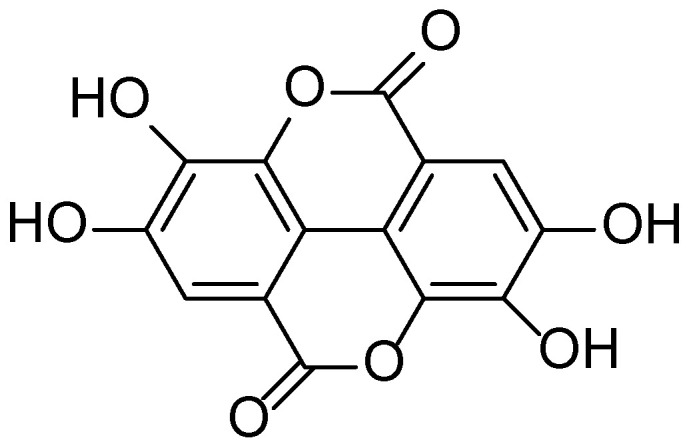
Structural formula of ellagic acid, the main biologically active compound in *Rubus* spp.

**Figure 9:**
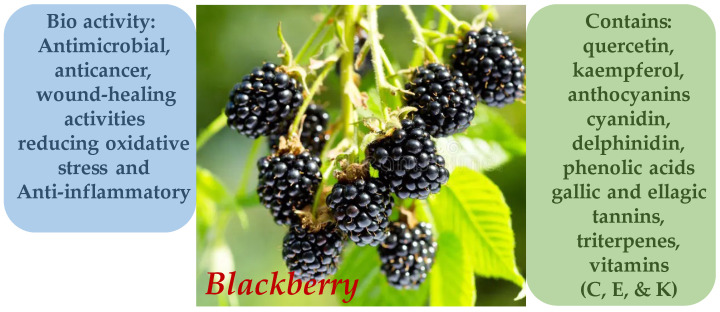
Phytotherapeutic application and chemical composition of *Rubus vulgaris*.

The phytochemical profile of *R. vulgaris* includes a wide array of bioactive compounds such as flavonoids (quercetin, kaempferol), anthocyanins (cyanidin, delphinidin), phenolic acids (gallic and ellagic) (Figure 8), tannins, triterpenes, and vitamins (C, E, and K). Of particular interest is the high content of ellagitannins and anthocyanins, which possess strong antioxidant and anti-inflammatory properties (Figure 9). Recent studies have shown that *R. vulgaris* extracts exhibit antimicrobial, anticancer, and wound-healing activities, primarily attributed to the presence of these compounds. For example, anthocyanins contribute to the neutralization of free radicals, thereby reducing oxidative stress and inflammatory responses in tissues [51,52,53,54].

In traditional medicine across Europe and Asia, the leaves and roots of blackberry (*Rubus vulgaris*) have been used to treat diarrhea, oral inflammations, skin disorders, and wounds. Decoctions and infusions were commonly applied as astringent and anti-inflammatory remedies for gastrointestinal and dermatological conditions. Ethnobotanical studies conducted in various regions confirm the widespread use of *Rubus vulgaris* in folk medicine for the treatment of skin diseases, highlighting its potential application in modern dermatological practice [55,56].

### 3.7. Common Dandelion (Taraxacum officinale)

*Taraxacum officinale*, a member of the Asteraceae family, is a widely distributed perennial herbaceous plant found across various climatic zones of Europe, Asia, North America, and Central Asia. In the territories of Kazakhstan, Russia, and Uzbekistan, dandelion commonly grows in meadows, along roadsides, in pastures, and urban areas, demonstrating high ecological adaptability and capacity for vegetative reproduction [56,57]. Due to its ease of collection and resilience to anthropogenic impacts, *T. officinale* is actively employed in both traditional and official phytotherapy.

**Figure 10:**
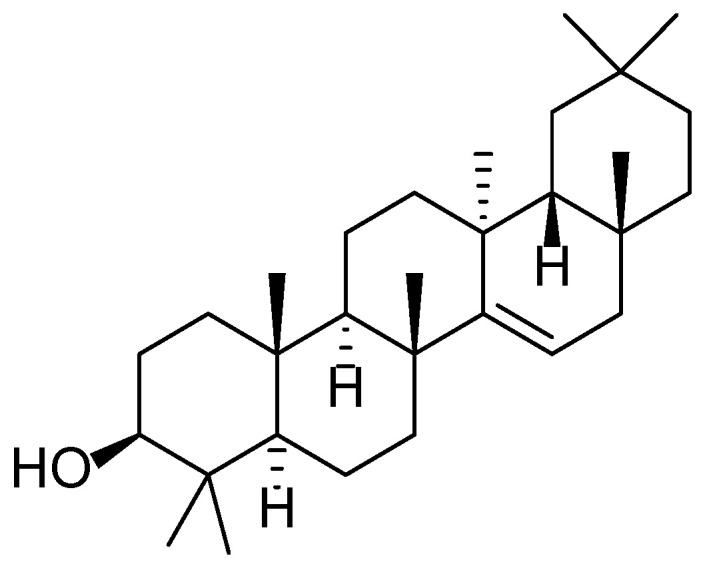
Structural formula of taraxerol—one of the major triterpenoids found in *Taraxacum officinale*.

The phytochemical composition of *Taraxacum officinale* is rich in bioactive compounds, including triterpenoids (taraxerol, taraxasterol) (Figure 10), flavonoids (luteolin, apigenin), phenolic acids (chlorogenic, caffeic, p-coumaric), inulin, bitter sesquiterpene lactones, as well as vitamins A, C, and E [58,59]. Of particular interest are hydroxycinnamic acids such as chlorogenic and cynarin, which exhibit pronounced antioxidant activity. The plant also contains saponins and mucilaginous substances that contribute to its emollient and anti-inflammatory effects. The antibacterial properties of *T. officinale* extracts are attributed to the synergistic action of flavonoids and phenolic acids, which inhibit the growth of pathogenic microorganisms, including *Staphylococcus aureus* and *Escherichia coli* [60,61].

In traditional medicine systems of Europe, China, and Central Asia, the leaves, roots, and latex of *Taraxacum officinale* have long been used for the treatment of dermatological conditions, including eczema, furunculosis, acne, and inflammatory skin eruptions. In Tibetan and Uyghur medicine, the root of the plant is valued for its cooling, detoxifying, and wound-healing properties. In Ayurveda, *T. officinale* is considered a *pitta*-reducing herb, believed to purify both the skin and blood. Recent pharmacological studies confirm that dandelion extracts are capable of suppressing pro-inflammatory markers (TNF-α, IL-6), enhancing antioxidant enzymes (SOD, CAT), and promoting skin regeneration [62,63].

Ethnobotanical surveys conducted in the southern regions of Kazakhstan and Kyrgyzstan have documented the use of fresh juice or compresses made from crushed *T. officinale* leaves for the treatment of wounds, inflammations, and insect bites. In certain localities, the plant is also applied in the form of ointments and infusions for topical use in skin disorders. These practices highlight a longstanding folk tradition of employing dandelion as an accessible remedy for inflammatory and infectious skin conditions, underscoring its potential as a valuable component in dermatological phytotherapeutics [64].

### 3.8. Common Flax (Linum usitatissimum)

*Linum usitatissimum* is one of the oldest cultivated crops, historically grown for both its fiber and oil. The plant exhibits high adaptability to a range of climatic conditions, which has facilitated its cultivation in more than 60 countries worldwide, including Canada, China, India, the United States, and most European nations. The most productive regions for flax cultivation are the Canadian Prairies (Manitoba, Saskatchewan, Alberta) and central India, where it is primarily grown as an oilseed crop. In Europe, flax is traditionally cultivated for fiber production, particularly in northwestern regions such as France, Belgium, and the Netherlands. The taxonomy of flax includes various forms—fiber flax, intermediate, and oilseed types—that differ morphologically and agronomically. All cultivated forms are believed to originate from the wild species *Linum angustifolium* (syn. *L. bienne*), domesticated independently in multiple regions since the Neolithic era. Due to its ecological plasticity, flax has successfully adapted to both temperate and warm climates. Its seeds and oil are widely utilized in food, pharmaceutical, and industrial applications [65,66].

**Figure 11:**
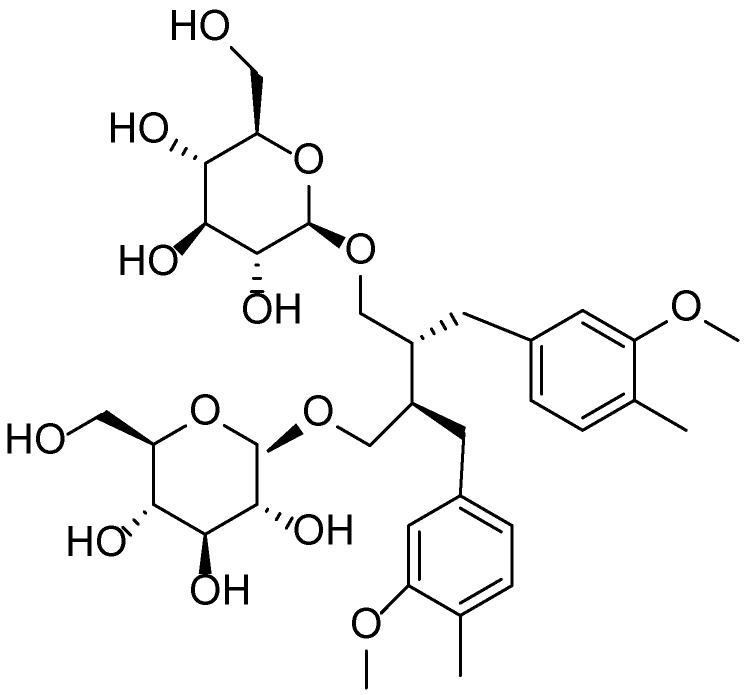
Structural formula of secoisolariciresinol diglucoside, the main lignan found in *Linum usitatissimum*.

*Linum usitatissimum* seeds (commonly known as flaxseed) possess a rich chemical profile, comprising proteins, lipids, dietary fibers, minerals, and biologically active compounds. Studies have shown that partial defatting and roasting of flaxseeds significantly increase the levels of crude protein, ash, and essential minerals such as potassium, magnesium, and iron, while enhancing functional properties like foaming capacity and water absorption [67]. Additionally, flaxseeds contain a substantial quantity of phenolic compounds, including lignans (notably secoisolariciresinol diglucoside, as shown in Figure 11), tannins, and flavonoids, which exhibit antibacterial activity against Gram-negative bacteria such as *Escherichia coli* and *Salmonella typhimurium* [68]. Germination of flaxseeds leads to a decrease in oil content and an increase in ascorbic acid and carotenoid levels; however, it also results in a reduction of total phenolic acids and oil stability, potentially affecting antioxidant capacity [69]. These characteristics underscore the value of *Linum usitatissimum* as a raw material in the food, pharmaceutical, and cosmetic industries.

*Linum usitatissimum* (flaxseed) has been widely used in traditional medicine for the treatment of skin disorders due to its anti-inflammatory, antioxidant, and regenerative properties. Studies have demonstrated that topical application of 5% and 10% flaxseed-based gel significantly accelerates the healing of full-thickness skin wounds in rats by promoting collagen synthesis, fibroblast proliferation, and vascularization [6]. Moreover, in horses exhibiting hypersensitivity to *Culicoides* bites, dietary supplementation with flaxseed reduced the affected skin area and lowered the levels of long-chain saturated fatty acids in hair, indicating an anti-inflammatory effect [70]. In Turkish folk medicine, flaxseed is traditionally employed in the form of decoctions, ointments, and poultices to treat burns, eczema, hemorrhoids, and other skin conditions, as well as to facilitate the maturation of purulent wounds [71]. These findings support the therapeutic potential of *Linum usitatissimum* in dermatological applications, although further clinical research is necessary to optimize its use.

### 3.9. European Cranberrybush (Viburnum opulus)

*Viburnum opulus* is a perennial shrub belonging to the family *Adoxaceae*, widely distributed across Europe, North Africa, and Asia. In both traditional and official medicine, various parts of the plant—including the bark, fruits, and flowers—are used due to their rich chemical composition and diverse pharmacological properties [72,73].

**Figure 12:**
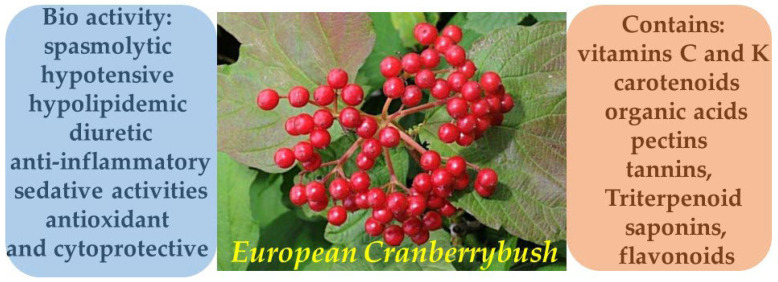
Phytotherapeutic application and chemical composition of *Viburnum opulus*.

The bark of *V. opulus* contains tannins, triterpenoid saponins, organic acids, and flavonoids. The fruits are rich in vitamins C and K, carotenoids, organic acids, and pectins. This composition contributes to the plant’s broad therapeutic effects, including spasmolytic, hypotensive, hypolipidemic, diuretic, anti-inflammatory, and sedative activities (Figure 12) [74,75,76].

**Figure 13:**
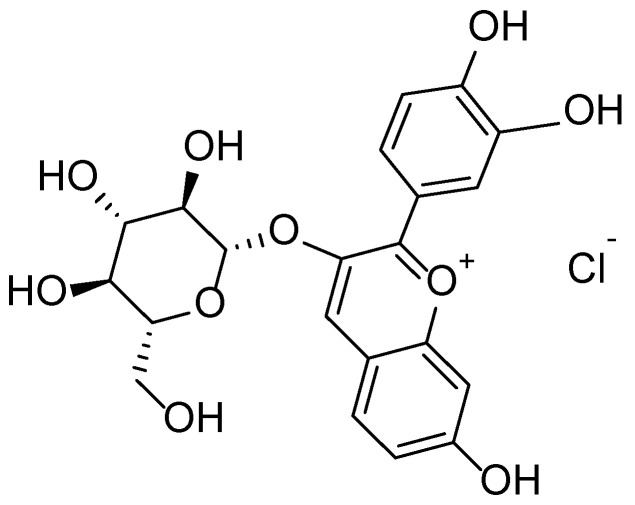
Structural formula of cyanidin-3-glucoside—a major anthocyanin found in *Viburnum opulus*.

*Viburnum opulus L.* exhibits high potential for dermatological applications, owing to the wide array of bioactive compounds found in its various morphological parts. According to a review by Kajszczak et al. [77], the fruits, bark, and leaves of *V. opulus* are rich in phenolic compounds, including flavonols (rutin, isorhamnetin), anthocyanins (cyanidin-3-glucoside) (Figure 13), catechins, and procyanidins. These constituents are responsible for the plant’s antioxidant, anti-inflammatory, and cytoprotective properties, which are particularly relevant in the treatment of inflammatory and age-related skin conditions.

*Viburnum opulus* extracts have been shown to effectively inhibit inflammatory responses mediated by reactive oxygen species and proinflammatory cytokines. A study by Moldovan et al. [78] demonstrated that silver nanoparticles synthesized using *V. opulus* fruit extract exhibit significant anti-inflammatory activity both in vitro (in UV-irradiated HaCaT keratinocyte cultures) and in vivo (in an acute inflammation model in rats). These findings suggest the potential use of *V. opulus*-derived compounds in topical formulations for the treatment of dermatological inflammation, including sunburn, eczema, and dermatitis.

A pharmacological study conducted by Khvorost et al. [79] further confirmed the suitability of *V. opulus* fruits for application in the cosmetic industry. Extracts obtained from the fruits exhibit antiseptic, moisturizing, whitening, and astringent properties, making them suitable for inclusion in masks, toners, creams, and soaps. Additionally, the phenolic constituents of *V. opulus* have been shown to promote skin regeneration and reduce oxidative stress induced by ultraviolet radiation. The study also noted that aqueous and ethanolic extracts contribute to improved skin condition by inhibiting matrix-degrading enzymes such as matrix metalloproteinase-1 (MMP-1) and reducing the production of interleukin-6 (IL-6) and tumor necrosis factor-alpha (TNF-α).

### 3.10. Garden Angelica (Angelica archangelica)

*Angelica archangelica*, also known as garden angelica, is a perennial herbaceous plant belonging to the family *Apiaceae*. It has been traditionally used in both European and Eastern medicine and is widely distributed across the northern and temperate regions of Europe and Asia. In medicine and cosmetology, the primary parts used are the roots and rhizomes, which possess a broad range of pharmacological properties due to their complex chemical composition [80,81].

The rhizomes of *A. archangelica* contain essential oils rich in furanocoumarins, sesquiterpene lactones, organic acids, tannins, and flavonoids. These compounds confer antibacterial, anti-inflammatory, spasmolytic, and antiseptic properties to the plant. Studies have demonstrated that angelica extracts can inhibit inflammatory processes by suppressing key pro-inflammatory mediators such as interleukin-1β and tumor necrosis factor-alpha (TNF-α). In addition, furanocoumarins exhibit photosensitizing activity, which is therapeutically applied in dermatology for the treatment of psoriasis and vitiligo within the framework of PUVA (psoralen + UVA) therapy [82,83,84,85].

*Angelica archangelica* also exhibits high antioxidant potential, making it a promising ingredient in skincare formulations aimed at protecting the skin from oxidative stress and premature aging. Extracts from the leaves and stems have demonstrated significant inhibition of lipid peroxidation (up to 93.9%) and strong free radical scavenging activity (DPPH, ABTS, DMPD), comparable to standard antioxidants such as α-tocopherol and Trolox. Furthermore, the extracts are rich in phenolic and flavonoid compounds known for their protective and membrane-stabilizing effects on skin cells. The mineral composition of the plant—including magnesium, calcium, phosphorus, and zinc—also contributes to the reinforcement of the skin barrier, regulation of inflammatory responses, and stimulation of tissue regeneration. The combined action of antioxidants and minerals makes *Angelica archangelica* a valuable botanical resource for the development of natural products intended for aging, sensitive, and damaged skin [86,87].

**Figure 14:**
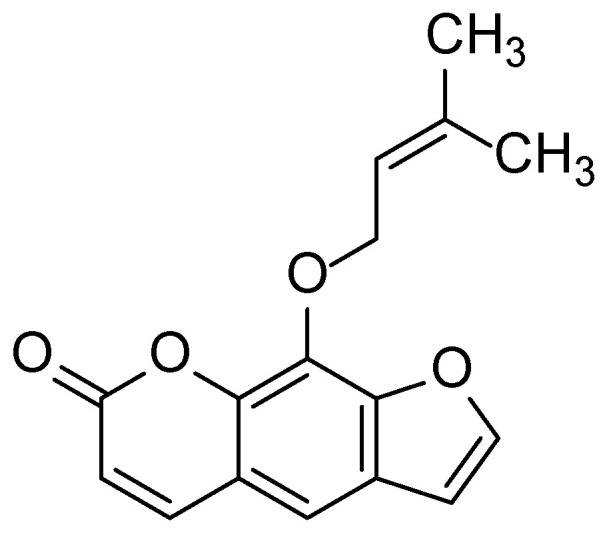
Structural formula of imperatorin—a major furanocoumarin component found in *Angelica archangelica*.

**Figure 15:**
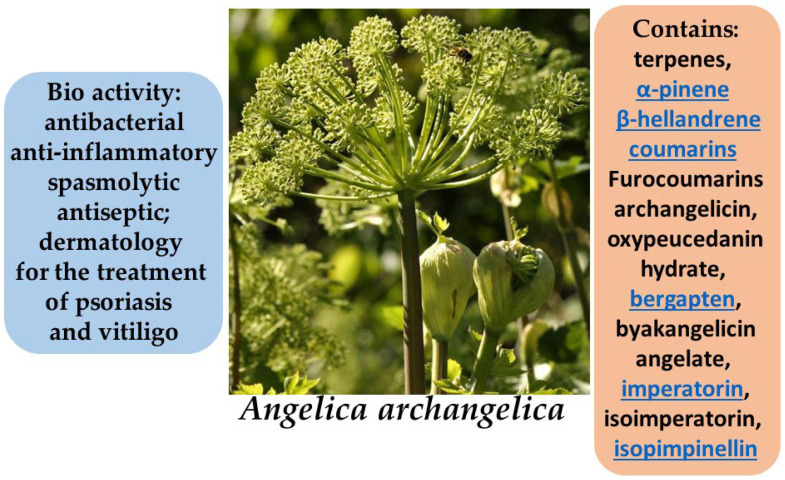
Phytotherapeutic application and chemical composition of *Angelica archangelica*.

Plant extracts, particularly root essential oils, have been shown to suppress the expression of pro-inflammatory cytokines (e.g., interleukin-6), the activity of the NF-κB transcription factor, and the generation of reactive oxygen species (ROS), thereby protecting the skin from oxidative and inflammatory damage. In addition, the essential oils of *Angelica archangelica* have demonstrated the ability to enhance skin permeability to active compounds, highlighting their potential use in transdermal drug delivery systems. Imperatorin, a key furanocoumarin (Figure 14) component of *A. archangelica* essential oil, plays a central role in these effects (Figure 15).

Due to these properties, components of *Angelica archangelica* are increasingly incorporated into creams, serums, lotions, and other cosmetic formulations aimed at skin restoration, protection, and rejuvenation [88].

### 3.11. Greater Burdock (Arctium lappa)

*Arctium lappa* L., a member of the Asteraceae family, is a widely distributed biennial plant native to Europe, Asia, and North America. It typically grows in temperate climates and is commonly found along roadsides and in wastelands, abandoned gardens, and other disturbed habitats. The plant is cultivated in East Asia (notably China and Japan) and several European countries for both medicinal and nutritional purposes [89,90,91].

**Figure 16:**
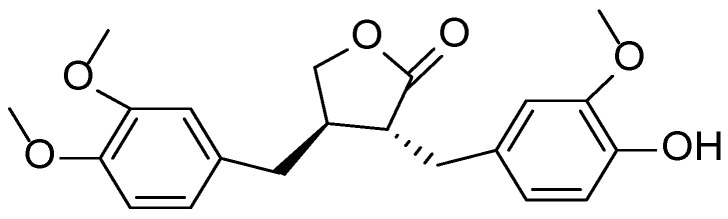
Structural formula of arctigenin, the main biologically active lignan found in *Arctium lappa*.

The phytochemical profile of *Arctium lappa* includes polyphenolic compounds, lignans (arctiin and arctigenin) (Figure 16), inulin, sesquiterpene lactones, phenolic acids (such as chlorogenic and caffeic acids), and essential oils. Recent studies highlight the pharmacological relevance of arctigenin and its derivatives, which exhibit significant antioxidant, anti-inflammatory, and anticancer properties. Lignans derived from burdock have demonstrated the ability to inhibit pro-inflammatory cytokine production and exert protective effects on skin cells [92,93,94].

In folk medicine, *Arctium lappa* is widely recognized as an effective remedy for inflammatory skin conditions such as dermatitis, eczema, and acne, as well as for burns, purulent lesions, and metabolic disorders. In Traditional Chinese and Japanese Medicine, burdock root is used for its detoxifying, anti-inflammatory, and wound-healing properties. Ethnobotanical studies from Eastern Europe also document the traditional use of *A. lappa* in the form of ointments, infusions, and compresses for the treatment of skin inflammation, wounds, and acne. Its pronounced biological activity highlights the potential of burdock for applications in dermatology and cosmetology [95,96].

### 3.12. Inula helenium L.

*Inula helenium* L., commonly known as elecampane, is a perennial herbaceous plant from the Asteraceae family, predominantly found in the temperate zones of Europe and Asia. In the wild, it is widespread across Eastern and Central Europe, the Caucasus, Western Siberia, Central Asia, and China. In Russia, *I. helenium* is broadly distributed in the European part, particularly in forested and forest-steppe regions, as well as in the Urals and southern Siberia. The plant favors moist soils rich in organic matter and typically grows along forest edges, riverbanks, wet meadows, and near marshes. While it tolerates partial shade, it thrives best in open, sunlit areas. Due to its recognized medicinal properties, *Inula helenium* is also cultivated in various regions as a valuable medicinal crop [97,98].

The primary medicinal parts of the plant are its roots and rhizomes, which are rich in a complex array of biologically active compounds, making the species a promising candidate for dermatological applications, particularly in the treatment of inflammatory and infectious skin conditions [99].

**Figure 17:**
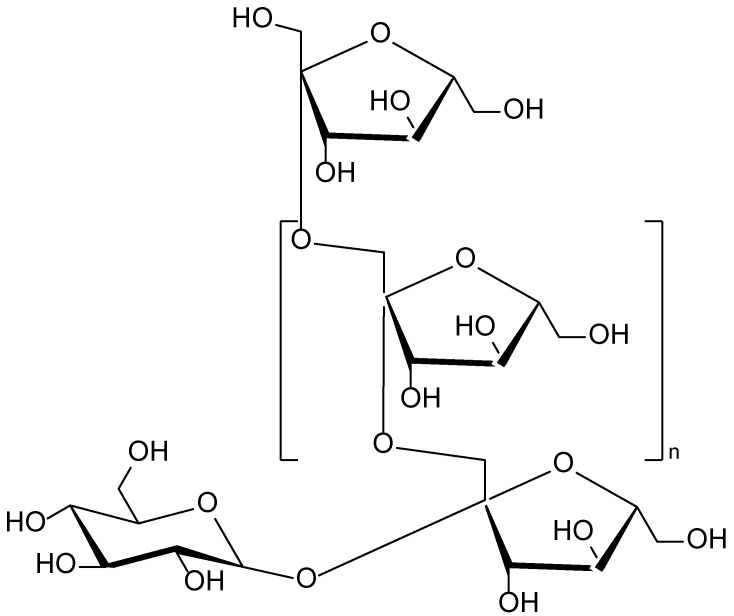
Structural formula of inulin—a natural fructan polysaccharide found in the roots of *Inula helenium*.

The root of *I. helenium* contains a high concentration of inulin (Figure 17)—a naturally occurring polysaccharide from the fructan group—which represents a significant portion of the plant’s carbohydrate reserves [100]. Inulin not only enhances the plant’s nutritional value but also exhibits notable prebiotic activity, contributing to the maintenance of the skin and mucosal microbiota. Additionally, literature reports indicate that inulin may strengthen skin barrier functions and exert anti-inflammatory effects, thereby positioning it as a promising component in the development of formulations for sensitive and damaged skin. The presence of inulin in combination with other bioactive constituents of *I. helenium*, such as sesquiterpene lactones and essential oils, further enhances its dermatological value [101].

Flavonoids and phenolic acids present in *Inula helenium*—including quercitrin, isoquercitrin, ferulic acid, and caffeic acid—contribute to the stabilization of cell membranes, enhancement of microcirculation, and protection of tissues against oxidative stress. These compounds exhibit antioxidant activity, inhibit lipid peroxidation, and protect epidermal cells from damage induced by ultraviolet radiation and reactive oxygen species [102,103].

Topical application of *Inula helenium*-based preparations has been shown to reduce skin swelling, erythema, and itching, making them beneficial in the treatment of eczema, dermatitis, psoriasis, and allergic skin eruptions. Furthermore, *I. helenium* extracts have been reported to accelerate reparative processes by stimulating fibroblast proliferation and collagen synthesis, thus positioning the plant as a valuable ingredient in wound-healing ointments and creams, particularly for chronic ulcers, burns, and abrasions [104].

In cosmetic practice, *Inula helenium* is incorporated into cleansing and toning formulations for skin prone to inflammation, irritation, and breakouts. Infusions and extracts are used in lotions, masks, and creams that help even skin tone, reduce oiliness, cleanse pores, and alleviate microinflammation. Decoctions of *I. helenium* are also applied in scalp care to strengthen hair, relieve itching, and reduce dandruff [105].

### 3.13. Marshmallow (Althaea officinalis)

*Althaea officinalis* is a perennial herbaceous plant native to temperate regions of Europe, Western Asia, and North Africa. It typically grows in moist meadows, along riverbanks and lake shores, as well as in marshy lowlands [106].

**Figure 18:**
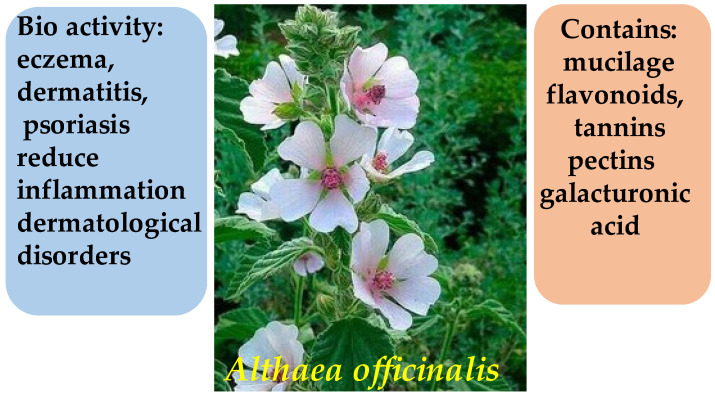
Phytotherapeutic application and chemical composition of *Althaea officinalis*.

**Figure 19:**
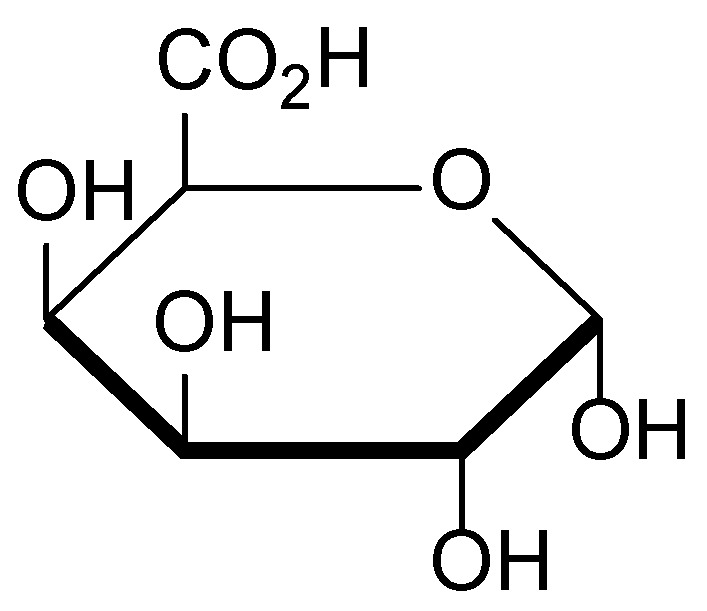
Structural formula of galacturonic acid, the principal structural unit of mucilaginous polysaccharides in *Althaea officinalis*.

The main biologically active constituents of *A. officinalis* include polysaccharides (mucilage), flavonoids, tannins, and pectins (Figure 18) [107]. The mucilage found in the roots of the plant, primarily composed of galacturonic acid (Figure 19)—the principal structural unit of mucilaginous polysaccharides—exhibits demulcent and emollient properties, making it effective in the treatment of inflammatory skin conditions [108]. Flavonoids and tannins contribute to its anti-inflammatory and antimicrobial activities, promoting wound healing and reducing skin irritation [109].

In dermatology, *Althaea officinalis* is used to treat various skin conditions, including eczema, dermatitis, and psoriasis [110]. Herbal preparations derived from marshmallow help reduce inflammation, accelerate tissue regeneration, and protect damaged skin from external irritants. Infusions and decoctions of marshmallow root are traditionally applied as compresses and poultices to relieve symptoms of dermatological disorders [111].

Pharmacological studies support the efficacy of *A. officinalis* in the treatment of skin diseases. The mucilage’s demulcent properties form a protective barrier on the skin surface, preventing dehydration and promoting wound healing. The anti-inflammatory activity of flavonoids and tannins helps alleviate inflammation and prevents secondary infections in damaged skin areas [112,113].

### 3.14. Narrow-leaved Lavender (Lavandula angustifolia)

*Lavandula angustifolia* is a perennial subshrub from the Lamiaceae family, naturally occurring in mountainous regions of the Mediterranean, ranging from Spain and France to Italy, primarily at altitudes above 1500 m above sea level [114]. Two main subspecies are widely recognized: ssp. *angustifolia*, native to the French and Italian Alps, and ssp. *pyrenaica* from the Pyrenees. The latter has been shown to possess a different essential oil composition and is considered less suitable for commercial use. Due to its high ecological plasticity and resistance to adverse environmental conditions, *L. angustifolia* is successfully cultivated in Europe, Asia, and North America, particularly in countries with temperate climates and well-drained soils [115].

**Figure 20:**
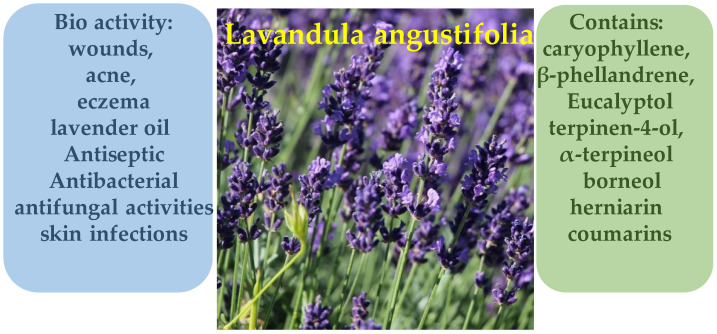
Phytotherapeutic application and chemical composition of *Lavandula angustifolia*.

The essential oil of *Lavandula angustifolia* is characterized by a complex and diverse chemical composition, including mono- and sesquiterpenoids, oxygenated compounds, and mineral elements. According to Jianu et al. [116], the primary constituents of the essential oil extracted from lavender flowers are caryophyllene (24.12%), β-phellandrene (16%), and eucalyptol (15.69%). Additionally, terpinen-4-ol (9.57%), α-terpineol (6%), and borneol (5.07%) were identified, indicating a high concentration of oxygenated monoterpenes (Figure 20). Another study reports that the composition of lavender essential oil may vary significantly depending on cultivation conditions and varietal differences [117]. Beyond terpenoids, the aerial parts of *L. angustifolia* have been found to contain compounds such as herniarin and coumarins, as well as a broad range of macro- and microelements, including Ca, Mg, Zn, Mn, Fe, Cu, and Na, further enriching the plant’s biochemical profile [118].

*Lavandula angustifolia* has long been used in traditional medicine due to its pronounced pharmacological properties. Extracts and essential oil derived from the plant are applied in the treatment of inflammatory and infectious skin diseases, as well as in the management of burns, wounds, acne, and eczema. According to Cardia et al. [119], the essential oil of *L. angustifolia* exhibits notable anti-inflammatory effects in both topical and systemic applications. In experimental animal models, the oil was shown to reduce edema, myeloperoxidase (MPO) activity, and nitric oxide (NO) production, indicating its ability to suppress acute inflammatory responses. Furthermore, as reported by Sharma et al. [120], lavender oil demonstrates antiseptic, antibacterial, and antifungal activities, making it effective against skin infections, including resistant strains such as *Staphylococcus aureus*. The plant also promotes tissue regeneration and wound healing. It is widely utilized in the form of creams, ointments, infusions, and essential oil preparations, including aromatherapy, as an adjunct treatment for dermatological and psychosomatic conditions.

**Figure 21:**
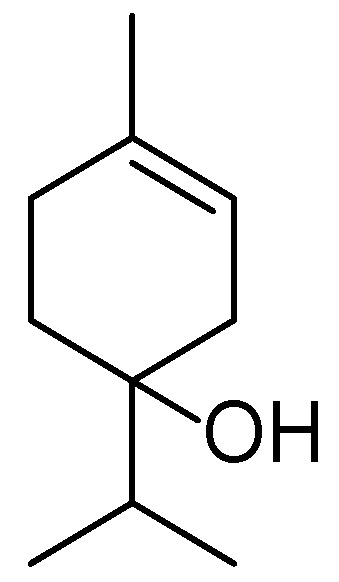
Structural formula of terpinen-4-ol—a key oxygenated monoterpene found in *Lavandula angustifolia* essential oil.

In cosmetology and dermatology, the essential oil of *Lavandula angustifolia* is actively employed for its anti-inflammatory, antibacterial, and antioxidant properties. Recent studies confirm its efficacy as a safe and natural skincare agent, particularly in depigmentation therapies. For instance, a study by Andrei et al. [121] demonstrated that a lavender-based cream reduced melanin levels in hyperpigmented skin areas by over one-third after two months of application. The key active compound, terpinen-4-ol (Figure 21), was identified as a potent tyrosinase inhibitor of the principal enzyme involved in melanogenesis—highlighting lavender oil as a promising natural depigmenting agent [121].

Additionally, as highlighted by Galea et al. [122], lavender ranks among the most sought-after essential oils in the dermo-cosmetic industry due to its soothing effects on irritated skin, along with its notable antibacterial and antiseptic properties. These attributes make it particularly valuable in skincare formulations for problematic skin, including acne, irritation, and inflammatory conditions.

It is also important to note that *Lavandula angustifolia* not only exerts beneficial effects on the skin but also promotes tissue regeneration and exhibits wound-healing properties, making it suitable for the treatment of burns and cutaneous infections. The study by Sharma et al. [120] emphasizes that lavender oil penetrates the skin rapidly, improves conditions such as eczema, psoriasis, and acne, and may also help prevent the formation of scar tissue. Thus, lavender occupies a prominent place among botanical ingredients in cosmetic formulations, owing to its comprehensive therapeutic profile.

### 3.15. Red Clover (Trifolium pratense L.)

*Trifolium pratense* L., commonly known as red clover, is a perennial herbaceous plant belonging to the Fabaceae family. It is widely distributed across temperate and subtropical regions of both hemispheres. In both wild and cultivated forms, it occurs in North America, Europe, northern China and Japan, southern Latin America, and Australasia, typically thriving in well-drained soils with neutral to slightly acidic pH (optimal range: 6.0–7.6) and moderate climates with annual precipitation exceeding 550 mm [123]. Historically, the plant has been cultivated in Europe since the 3rd century AD, and its widespread adoption in crop rotation during the 16th–17th centuries significantly contributed to agricultural development, particularly due to its nitrogen-fixing capacity. Today, *T. pratense* is actively grown in both organic and conventional farming systems worldwide, including in Russia, Japan, Canada, the United States, Brazil, and Australia [124].

**Figure 22:**
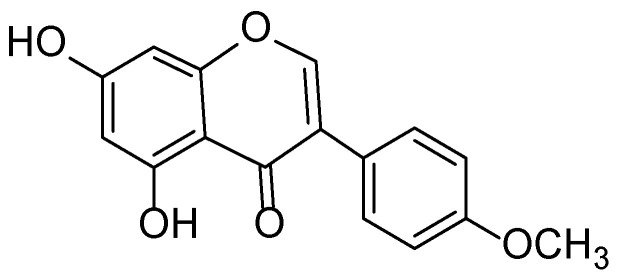
Structural formula of biochanin A—a major isoflavone found in *Trifolium pratense* L.

**Figure 23:**
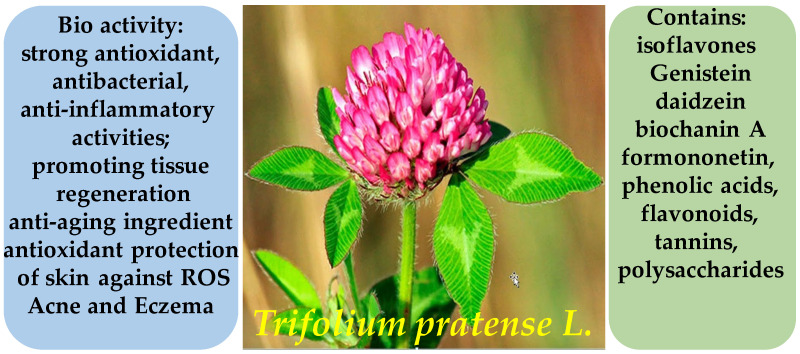
Phytotherapeutic application and chemical composition of *Trifolium pratense*.

Red clover is a valuable medicinal plant extensively used in phytotherapy and cosmetology due to its rich chemical composition and well-documented pharmacological properties. The main bioactive constituents of *T. pratense* extracts include isoflavones (genistein, daidzein, biochanin A (Figure 22), formononetin), phenolic acids, flavonoids, tannins, and polysaccharides (Figure 23). Isoflavones from red clover exhibit estrogen-like activity, making this plant particularly relevant for skincare formulations targeting aging skin affected by hormone-related changes, including decreased elasticity, dryness, and increased sensitivity [125,126].

In dermatology, *Trifolium pratense* (red clover) extracts have demonstrated notable efficacy as anti-inflammatory and wound-healing agents. A study by Farahpour et al. [127] revealed that topical application of a hydroethanolic extract of *T. pratense* on wounds in laboratory animals significantly accelerated the healing process. This was accompanied by active fibroblast proliferation, stimulation of collagen synthesis and re-epithelialization, as well as downregulation of pro-inflammatory and apoptotic markers. These findings highlight the therapeutic potential of red clover in the treatment of wounds, burns, and chronic dermatoses.

Additional value lies in combined phytotherapeutic formulations based on *T. pratense* and other medicinal plants. For instance, Antonescu et al. [128] developed topical preparations combining red clover and basil (*Ocimum basilicum*) extracts, which exhibited strong antioxidant, antibacterial, and anti-inflammatory activities. These compositions proved effective in restoring the skin barrier and promoting tissue regeneration, suggesting their potential use in managing inflammatory skin conditions such as acne and eczema.

In cosmetic applications, *T. pratense* is regarded as a promising anti-aging ingredient, particularly for postmenopausal women. Its isoflavones, acting as phytoestrogens, contribute to maintaining dermal structure and reducing the appearance of wrinkles. Furthermore, the plant’s phenolic compounds provide antioxidant protection to skin cells against damage induced by ultraviolet radiation and environmental stressors [129].

Due to its rich phytochemical composition and multifunctional properties, *Trifolium pratense* can be incorporated into a wide range of dermatological and cosmetic formulations, including creams, gels, masks, therapeutic ointments, and anti-aging products. Contemporary research supports its safety, biocompatibility, and high efficacy in topical applications.

### 3.16. Virginian Witch Hazel (Hamamelis virginiana)

*Hamamelis virginiana* is a deciduous shrub belonging to the family *Hamamelidaceae*, native to the eastern regions of North America. Its leaves and bark are widely utilized in medicine and cosmetology due to their rich content of biologically active compounds [130,131].

**Figure 24:**
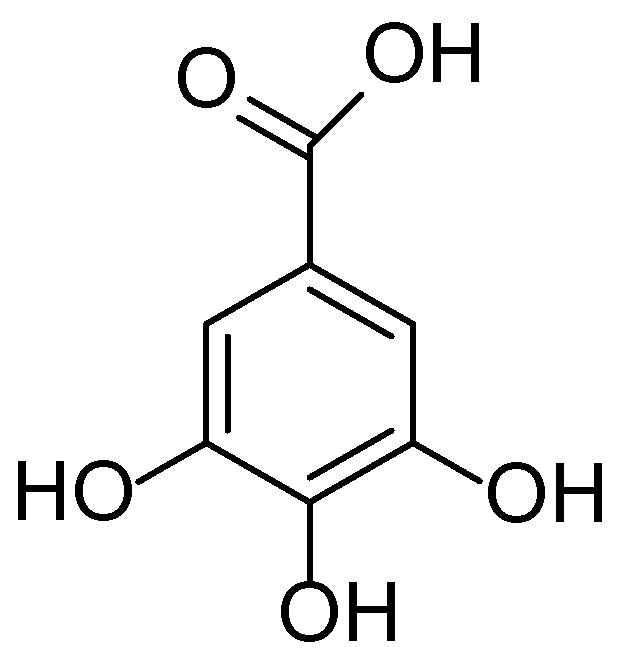
Structural formula of gallic acid—a major phenolic acid found in *Hamamelis virginiana*.

The leaves of *H. virginiana* contain significant levels of tannins (particularly hamamelitannin), flavonoids (including quercetin), gallic acid (Figure 24), and polysaccharides. The bark is especially rich in hydrolyzable tannins. This phytochemical composition underlies the plant’s astringent, anti-inflammatory, and antioxidant properties [132,133].

In dermatology, *Hamamelis virginiana* extract is used for the care of oily and problematic skin: it regulates sebum production, tightens pores, and helps prevent inflammation. Its anti-inflammatory properties reduce redness and irritation, making it particularly beneficial for sensitive skin. Moreover, witch hazel strengthens capillary walls, thereby diminishing the appearance of couperose (telangiectasia) [134,135].

In cosmetology, *H. virginiana* is incorporated into toners, creams, and cleansing formulations designed for problem skin, where it aids in reducing sebum secretion and minimizing pore size. Its vascular-strengthening effects are utilized in products aimed at treating couperose. In men’s skincare, witch hazel is commonly included in aftershave products for its soothing and restorative effects on the skin [136,137].

### 3.17. Smooth Brome (Bromus inermis Leyss.)

*Bromus inermis* Leyss. is a rhizomatous perennial grass belonging to the Poaceae family, native to Central and Eastern Europe and Western Asia. It has been widely naturalized in North America, Canada, Australia, and several Eastern European countries. Due to its adaptability to diverse climatic conditions and high drought resistance, *Bromus inermis* is extensively cultivated in temperate regions and thrives in steppe, meadow, and subalpine zones. It is commonly employed as a forage crop, a soil-stabilizing species, and a component in land reclamation efforts [138,139].

**Figure 25:**
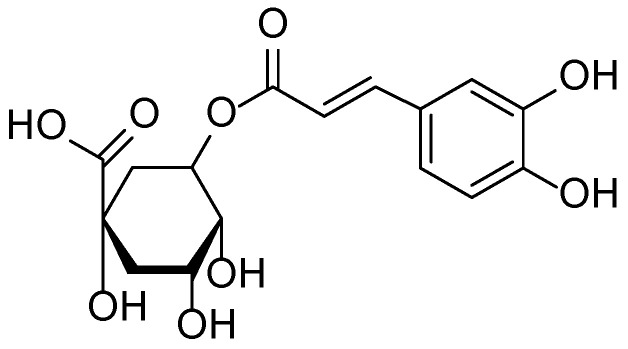
Structural formula of chlorogenic acid—a hydroxycinnamic acid found in *Bromus inermis*.

The phytochemical composition of *Bromus inermis* underpins its potential pharmacological activity. Analyses of aqueous and ethanolic extracts have revealed the presence of phenolic compounds (including phenolic acids and flavonoids), coumarins, essential oils, and structural polysaccharides derived from the plant cell wall [140]. Of particular interest is the detection of chlorogenic (Figure 25) and ferulic acids in the leaves and stems—bioactive molecules with well-established antioxidant and anti-inflammatory properties. These compounds have been shown to neutralize reactive oxygen species (ROS) and downregulate the expression of pro-inflammatory cytokines, which is of therapeutic relevance in treating dermatological conditions associated with oxidative stress and chronic inflammation.

Due to the demonstrated antibacterial and antifungal properties of *Bromus inermis* extracts, the plant is considered a potential source of bioactive compounds for dermatological and cosmetic applications. Studies have shown inhibition of pathogenic strains including *Staphylococcus aureus*, *Escherichia coli*, *Pseudomonas aeruginosa*, and *Candida albicans*, suggesting the possible incorporation of this species into topical formulations for the treatment of acne, seborrheic dermatitis, mycoses, and other infectious-inflammatory skin conditions [141].

Beyond its antimicrobial effects, *Bromus inermis* extracts exhibit regenerative and moisturizing potential, attributed to the presence of polysaccharides and humic-like substances. These properties support the use of the plant as a base for phytocosmetic compositions with restorative action, particularly for damaged epidermis, aging skin, and increased sensitivity. Additionally, the plant’s low allergenic potential makes it suitable for inclusion in hypoallergenic dermatological products. In veterinary medicine, *Bromus inermis* has also been explored as a therapeutic component in managing atopic skin conditions in animals caused by seasonal airborne allergens [142], further confirming its versatility and safety profile.

### 3.18. Turmeric (Curcuma longa)

*Curcuma longa* is a perennial herbaceous plant belonging to the family *Zingiberaceae*. It is native to the southwestern region of India, from where it has spread to various parts of Asia, Africa, and South America. Today, turmeric is cultivated across a broad geographic range, including India, China, Indonesia, Thailand, Sri Lanka, Bangladesh, and tropical regions of Africa and South America. India remains the world’s largest producer and exporter of turmeric [143,144]. Experimental studies have also demonstrated the successful cultivation of *Curcuma longa* in a variety of soil types, including dark-red, gray, and red soils in Okinawa (Japan), indicating the species’ strong adaptability to diverse agroecological conditions [145].

**Figure 26:**
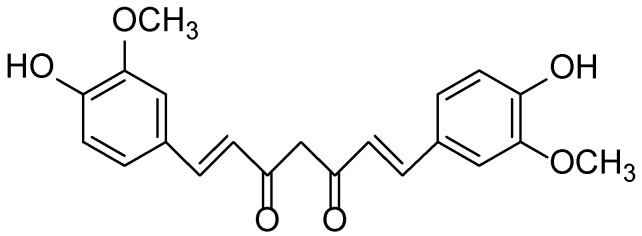
Structural formula of curcumin—the principal bioactive compound of *Curcuma longa*.

The chemical composition of *Curcuma longa* is highly diverse and comprises more than 235 identified compounds, the majority of which are phenolic and terpenoid metabolites. The principal bioactive constituents responsible for its pharmacological effects are curcuminoids (diarylheptanoids) and essential oils. Curcumin (C_21_H_20_O_6_) (Figure 26), demethoxycurcumin, and bisdemethoxycurcumin—the three primary curcuminoids found mainly in the rhizomes—constitute approximately 3–15% of the dry weight and exhibit well-documented anti-inflammatory, antioxidant, and anticancer activities [145]. The essential oil fraction, composed mainly of sesquiterpenoids and monoterpenes, varies in composition depending on geographical origin, cultivar, and cultivation conditions [146]. Notable constituents include ar-turmerone, α-turmerone, and β-turmerone, which serve as marker compounds for quality assessment and standardization of *Curcuma longa*-based products [147]. Additionally, GC-MS analysis of *C. longa* leaf essential oil revealed the presence of α-phellandrene, eucalyptol, and 2-carene, all of which possess antioxidant and antibacterial activities [148,149].

*Curcuma longa* has long held a significant position in traditional Asian medicine. It has been extensively used in Ayurvedic, Siddha, Chinese, and Tibetan systems for the treatment of various skin ailments, including wounds, burns, and infections, and as a cosmetic agent to improve complexion and skin health. Historically, turmeric rhizome powder was applied topically for skin purification, acne treatment, and inflammation reduction, as well as in the form of pastes and creams to combat photoaging and skin irritation [150,151].

Modern research validates these traditional applications and further elucidates the dermatological potential of *Curcuma longa*. For instance, it has been demonstrated that a hot-water extract of *Curcuma longa* (WEC) significantly suppresses inflammatory responses in UVB-irradiated human keratinocytes. The extract reduces the production of pro-inflammatory cytokines such as TNF-α and modulates the synthesis of hyaluronic acid, thereby improving skin hydration and barrier function. These findings support the systemic application of *C. longa* in skin protection and restoration [152].

Additionally, topical formulations of turmeric extract have been developed using liposomes, ethosomes, and transfersomes, which have shown enhanced hydration effects and seboregulation with prolonged use. These advanced delivery systems also provide photoprotective, antioxidant, and moisturizing benefits, establishing *Curcuma longa* as a promising ingredient for anti-aging and therapeutic cosmeceuticals [153].

### 3.19. White Willow (Salix alba)

*Salix alba*, commonly known as white willow, is a widely distributed deciduous tree species from the Salicaceae family, predominantly found in temperate regions. Its natural range spans across Europe, Western and Central Asia, and parts of North Africa. In the wild, *S. alba* typically grows along rivers and on lake shores, wet meadows, floodplains, and other moist habitats. It thrives particularly well in riparian zones, where it plays a crucial ecological role in bank stabilization and ecosystem support [154,155].

**Figure 27:**
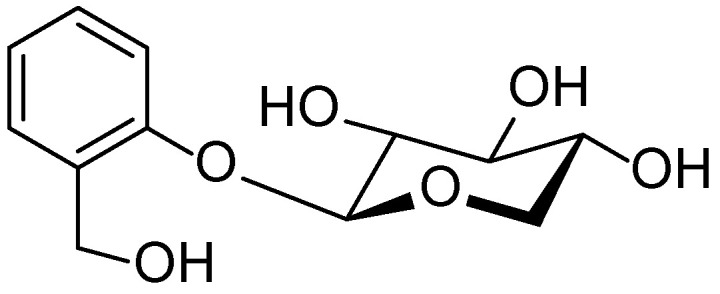
Structural formula of salicin—the primary phenolic glycoside found in *Salix alba*.

*Salix alba* L. is characterized by a rich chemical profile, comprising over 300 identified secondary metabolites, including phenolic glycosides (notably salicin), flavonoids (e.g., quercetin, rutin, catechins), organic acids, lignans, terpenoids, and simple phenolics. The primary bioactive compound—salicin (Figure 27)—is metabolized in the human body into salicylic acid, which exhibits pronounced anti-inflammatory and analgesic properties similar to those of aspirin. Studies have demonstrated that *S. alba* bark extract possesses not only strong antioxidant activity but also significant antimicrobial properties, including the inhibition of pathogenic bacteria such as *Escherichia coli* and *Staphylococcus aureus*, as well as fungi. This makes the plant a promising candidate for the development of topical formulations for the treatment of inflammatory and infectious skin diseases.

In addition, the phenolic compounds and flavonoids of *S. alba* have been shown to suppress the activity of pro-inflammatory cytokines (TNF-α, IL-6) and enzymes (COX-1, COX-2) involved in inflammatory pathways. These effects suggest its potential application in both dermatology and cosmetology, particularly in the treatment of acne, dermatitis, eczema, and photoaging of the skin [156,157,158].

The studies by L.M. Maloshtan and V.V. Pidgaina (2022) [159], as well as that of Edson L. Maistro et al. (2020) [160], demonstrated that *Salix alba* L. (white willow) holds significant potential for the treatment of dermatological conditions, particularly those of inflammatory and allergic origin. In preclinical investigations of a topical cream containing *S. alba* bark extract and zinc, conducted in a model of allergic contact dermatitis, a statistically significant reduction in IgE levels and neutrophil phagocytic activity was observed, indicating pronounced immunotropic effects. The cream showed efficacy comparable to or exceeding that of reference drugs, supporting its potential use in the comprehensive treatment of inflammatory and allergic skin conditions associated with pruritus, erythema, and impaired barrier function.

Additionally, Maistro et al. [160] reported that *Salix alba* bark extract—rich in phenolic constituents such as salicin, salicylic acid, salidroside, saligenin, and salicortin—exhibits potent antioxidant and anti-inflammatory activity. These compounds act synergistically to suppress inflammation mediated by proinflammatory cytokines. Although moderate genotoxic effects were observed at high concentrations in leukocyte cultures, such effects were absent in metabolically active systems (HepG2), confirming the relative safety of topical use. Therefore, *Salix alba* can be considered a promising botanical source for the development of topical preparations aimed at controlling inflammation, modulating cutaneous immune responses, and preventing the chronic progression of dermatoses.

### 3.20. White Wormwood (Artemisia terrae-albae)

*Artemisia terrae-albae* Krasch., a member of the Asteraceae family, is a perennial subshrub widely distributed across arid regions of Central Asia and Southern Europe. This plant thrives in dry steppe, semi-desert, and desert habitats characterized by poor soils and low moisture availability, reflecting its remarkable adaptability and resistance to extreme environmental conditions [161,162,163].

**Figure 28:**
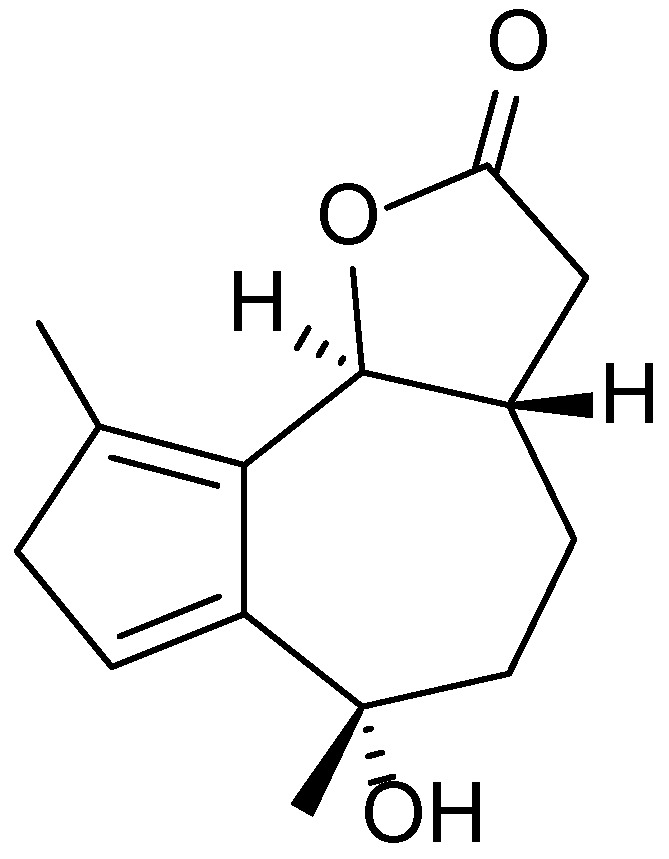
Structural formula of artabsin, the main biologically active sesquiterpene lactone found in *Artemisia terrae-albae*.

The chemical composition of *A. terrae-albae* includes a wide range of biologically active constituents, most notably terpenoids (mono- and sesquiterpenes), flavonoids (such as kaempferol, quercetin, and luteolin), phenolic acids (including chlorogenic, caffeic, and gallic acids), coumarins, and essential oils. Recent studies have demonstrated the plant’s antioxidant, anti-inflammatory, antibacterial, antifungal, and wound-healing properties, which are attributed to these bioactive compounds. Sesquiterpene lactones—such as artabsin (Figure 28) and tauremizine—exhibit particularly potent biological activity, effectively suppressing inflammatory responses and promoting the regeneration of damaged tissues [164,165,166].

In traditional medicine of Central Asia and Eastern Europe, *Artemisia terrae-albae* is used to treat skin disorders such as dermatitis, eczema, and psoriasis, as well as inflammatory conditions, wounds, and burns. In Traditional Chinese Medicine, various *Artemisia* species are recognized for their applications in treating inflammation and bacterial and fungal infections, and are commonly included in medicinal baths and topical ointments. Ethnobotanical studies conducted in Kazakhstan and Uzbekistan confirm the widespread use of *A. terrae-albae* in the form of decoctions, infusions, and topical extracts, highlighting its potential for the development of modern pharmaceutical formulations with pronounced antibacterial and anti-inflammatory properties [167,168,169].

## 4. Perspectives for Investigations

The medicinal plants examined in this study present substantial potential for integration into therapeutic applications targeting various dermatological conditions (Table 1). Detailed phytochemical analysis emphasizes the presence of bioactive constituents, notably flavonoids, anthocyanins, phenolic acids, tannins, sesquiterpenes, and polysaccharides, each recognized for their potent antioxidant, anti-inflammatory, antimicrobial, and wound-healing properties. Such pharmacological actions substantiate traditional ethnomedicinal practices, supporting their efficacy and relevance in modern dermatological formulations.

The comprehensive assessment covers species including *Rubus vulgaris, Plantago major*, *Artemisia terrae-albae*, *Eryngium planum*, *Angelica archangelica*, *Bergenia crassifolia*, *Arctium lappa*, *Hamamelis virginiana*, *Inula helenium*, *Linum usitatissimum*, among others, traditionally utilized in managing dermatological disorders like eczema, psoriasis, acne, and dermatitis. The contemporary scientific literature confirms the therapeutic value of these plants, elucidating mechanisms involving modulation of pro-inflammatory cytokine production, inhibition of inflammatory enzymes (such as COX-2), and enhancement of skin regeneration and barrier repair processes.

For instance, *Plantago major* contains aucubin, a bioactive iridoid glycoside renowned for its pronounced anti-inflammatory and regenerative capabilities. Similarly, *Rubus vulgaris* is notable for its high content of anthocyanins and ellagitannins, compounds that confer significant antioxidant and antimicrobial effects. *Artemisia terrae-albae*, rich in sesquiterpenes such as chamazulene, demonstrates substantial wound-healing and anti-inflammatory activities.

Integration of these medicinal plants into dermatological and cosmeceutical products offers an effective and safer alternative to synthetic therapeutics, which often present adverse effects upon prolonged use. However, several challenges remain to be addressed to ensure their clinical applicability. These include the need for standardization of active constituents, control of batch-to-batch variability, and establishment of pharmacokinetic and toxicological profiles. Despite the long history of use, rigorous clinical trials are still lacking for most plant-based dermatological agents, limiting their evidence-based validation. Furthermore, the complex regulatory landscape in different countries hinders the formal approval of herbal preparations, requiring harmonized guidelines on quality, safety, and efficacy.

To facilitate broader clinical acceptance, future research should focus on harmonizing extraction protocols, developing standardized formulations, and generating robust clinical data through well-designed studies.

## 5. Conclusions

This comprehensive review demonstrates that numerous medicinal plants traditionally used in dermatological applications possess a wide range of pharmacologically active constituents, including flavonoids, phenolic acids, tannins, and sesquiterpenes. These compounds exhibit clinically relevant antioxidant, anti-inflammatory, antimicrobial, and wound-healing properties, supporting both historical ethnomedical practices and emerging evidence from modern pharmacological studies. The reviewed species, particularly those from Central Asian traditions, represent promising candidates for the development of novel dermatological and cosmeceutical formulations.

However, their clinical implementation requires addressing several challenges, including variability in plant material, lack of standardization protocols, and limited clinical validation. Future research should prioritize the development of reproducible extraction methods, precise phytochemical profiling, and well-designed clinical trials to confirm therapeutic efficacy and safety. In addition, identification of lead compounds, assessment of potential adverse effects and herb–drug interactions, and compliance with regulatory standards are essential steps toward translating traditional plant-based therapies into evidence-based dermatological practice.

## Figures and Tables

**Table 1:** Phytochemical composition and therapeutic uses of medicinal plants for dermatological conditions.

No.	Plant Species	Key Bioactive Compounds	Pharmacological Activities	Dermatological Applications	References
1.1	*Bergenia crassifolia*	Arbutin, tannins, flavonoids	Anti-inflammatory, depigmenting, antimicrobial	Burns, hyperpigmentation, inflammation	[13,14,15,16,17,18,19]
1.2	*Sambucus nigra*	Flavonoids, anthocyanins, organic acids	Antioxidant, anti-inflammatory, antibacterial	Inflammation, anti-photoaging	[22,23,24,25,27]
1.3	*Plantago major*	Aucubin, flavonoids, phenolic acids	Anti-inflammatory, wound-healing, antimicrobial	Wounds, burns, eczema	[31,32,33,34,35]
1.4	*Solidago canadensis*	Flavonoids, saponins, essential oils	Anti-inflammatory, antioxidant, antimicrobial	Inflammation, eczema	[36,37,38,39]
1.5	*Tussilago farfara*	Flavonoids, phenolic acids, sesquiterpenes	Anti-inflammatory, antioxidant, antimicrobial	Skin irritations, acne, wounds	[42,43,44,45,46]
1.6	*Rubus vulgaris*	Anthocyanins, ellagitannins, phenolic acids	Antioxidant, anti-inflammatory, antimicrobial	Eczema, psoriasis, acne	[51,52,53,54]
1.7	*Taraxacum officinale*	Sesquiterpenes, flavonoids, phenolic acids	Anti-inflammatory, antioxidant, antimicrobial	Eczema, acne, inflammation	[60,61,62,63,64]
1.8	*Linum usitatissimum*	Lignans, phenolic acids, fatty acids	Anti-inflammatory, antioxidant, wound-healing	Burns, eczema, dermatitis	[68,69,70,71]
1.9	*Viburnum opulus*	Flavonoids, anthocyanins, tannins	Antioxidant, anti-inflammatory, antimicrobial	Inflammation, eczema, dermatitis	[74,75,76,78,79]
1.10	*Angelica archangelica*	Furanocoumarins, sesquiterpene lactones	Anti-inflammatory, antioxidant, antibacterial	Psoriasis, inflammation, vitiligo	[82,83,84,85,86,87,88]
1.11	*Arctium lappa*	Lignans, phenolic acids, inulin	Anti-inflammatory, antioxidant, antimicrobial	Dermatitis, acne, wounds	[92,93,94,95,96]
1.12	*Inula helenium*	Inulin, sesquiterpene lactones, flavonoids	Anti-inflammatory, antioxidant, wound-healing	Eczema, dermatitis, psoriasis, wounds	[99,100,102,103,104,105]
1.13	*Althaea officinalis*	Polysaccharides, flavonoids, tannins	Anti-inflammatory, emollient, antimicrobial	Eczema, psoriasis, inflammation	[108,109,110,111,112,113]
1.14	*Lavandula angustifolia*	Terpenoids, coumarins, essential oils	Anti-inflammatory, antibacterial, antioxidant	Acne, eczema, inflammation	[119,120,121]
1.15	*Trifolium pratense*	Isoflavones, flavonoids, phenolic acids	Anti-inflammatory, antioxidant, antimicrobial	Aging skin, eczema, wounds	[125,126,127,128,129]
1.16	*Hamamelis virginiana*	Tannins, flavonoids, gallic acid	Astringent, anti-inflammatory, antioxidant	Oily skin, acne, inflammation	[132,133,136,137]
1.17	*Bromus inermis*	Phenolic acids, coumarins, essential oils	Anti-inflammatory, antioxidant, antimicrobial	Acne, dermatitis, inflammation	[141,142]
1.18	*Curcuma longa*	Curcuminoids, sesquiterpenoids, essential oils	Anti-inflammatory, antioxidant, antimicrobial	Acne, eczema, inflammation, anti-photoaging	[145,148,149,150,151,152,153]
1.19	*Salix alba*	Salicin, flavonoids, phenolic acids	Anti-inflammatory, antioxidant, antimicrobial	Acne, dermatitis, inflammation	[156,157,158,159,160]
1.20	*Artemisia terrae-albae*	Sesquiterpenes, flavonoids, phenolic acids	Anti-inflammatory, antioxidant, antimicrobial	Dermatitis, eczema, psoriasis, inflammation	[164,165,166,167,168,169]

## Data Availability

Not applicable.
